# Anxiety-related impairment of inhibition during early sensory processing: an MMN/MOR study

**DOI:** 10.1038/s41598-026-49460-9

**Published:** 2026-04-29

**Authors:** Tomokazu Urakawa, Kentaro Imai, Keigo Mikami, Kyoko Shibata, Takuya Obata, Toshimi Okazaki, Osamu Araki

**Affiliations:** 1 Integrated Control System Development Division, Mazda Motor Corporation, 3-1 Shinchi, Fuchu-Cho, Aki-Gun, Hiroshima, 730-8670 Japan; 2 Department of Applied Physics, Tokyo University of Science, 6-3-1 Niijuku, Katsushika-Ku, Tokyo, 125-8585 Japan

**Keywords:** Attentional control theory, Trait anxiety, Mismatch negativity, Mismatch oscillatory response, Neuroscience, Psychology, Psychology

## Abstract

When a behavioral task becomes attentionally demanding, anxiety-prone individuals become more susceptible to task-irrelevant sensory events. Attentional control theory proposes that this susceptibility arises from impairments in the inhibition function, a central executive function that regulates responses to sensory stimuli. This study examined whether anxiety-related impairment of inhibition affects early sensory processing of task-irrelevant auditory stimuli, as measured by mismatch negativity (MMN), an early pre-attentive electroencephalographic response evoked by abrupt auditory changes. MMNs were recorded using an oddball paradigm while participants performed a visual task of varying difficulty. The results showed that the enhancement of MMN to a task-irrelevant high-frequency tone at a latency within 250 ms was significantly associated with trait anxiety when the visual task became more attentionally demanding, suggesting that impaired inhibition intrudes on early sensory processing. Furthermore, time–frequency analyses revealed that power enhancements of mismatch oscillatory responses in the alpha/low-beta band, occurring approximately up to 450 ms after stimulus onset, were significantly correlated with trait anxiety under high attentional demand. These findings suggest that compensatory top-down mediated sensory suppression of task-irrelevant events occurs through early sensory processing, along with impaired distractor inhibition reflected in MMN. This study proposes that impaired inhibition and compensatory sensory suppression in anxiety-prone individuals occur in a multi-tiered manner, even at the pre-attentive level.

**Supplementary Information:**

The online version contains supplementary material available at 10.1038/s41598-026-49460-9.

## Introduction

In an ever-changing sensory environment, the brain inevitably receives a vast amount of incoming sensory input, regardless of whether the input is relevant to a behavioral goal. Attentional control theory (ACT) argues that individuals with a predisposition to anxiety have an imbalance between goal-directed and stimulus-driven attentional systems, where the former has decreased influence and the latter has increased influence^1,2^. Susceptibility to sensory changes in anxiety-prone individuals has been reported in various experimental tasks such as anti-saccade^3^ and perceptual load tasks^4^. The adverse effects of anxiety on susceptibility to sensory changes are thought to originate from impaired central executive functions involving attentional control^1^. In particular, one of the central executive function impaired in anxiety-prone individuals is inhibitory processing^1,2^, which resists disruption from sensory changes while maintaining the current task goal. Such anxiety-related impairment of inhibition processing, presumed to be mediated by degraded top-down neural processing, is theoretically assumed to be modality-free and is expected to emerge during a behavioral task that is attentionally demanding.

Neural mechanisms underlying impaired inhibition in anxiety-prone individuals have been reported^5–7^. Functional magnetic resonance imaging (fMRI) studies^5,6^ have shown altered neural activity in individuals with high trait anxiety within the dorsolateral prefrontal cortex (DLPFC), a region critical for executive control^8^. These alterations in the DLPFC were interpreted as reduced recruitment of attentional control or decreased neural processing efficiency. Using the color-word Stroop task^6^, researchers further reported decreased functional connectivity between the DLPFC and other task-relevant cortical areas in high-anxiety individuals during incongruent trials compared with congruent trials, suggesting that reduced connectivity impairs inhibitory processing of task-irrelevant stimulus features.

An important but unresolved question is whether anxiety-related impaired inhibition, originating from degraded executive function, extends to early sensory processing following the onset of task-irrelevant stimuli. A prior electroencephalographic (EEG) study investigated this question by examining the ability to filter out task-irrelevant salient stimuli through early sensory processing^9^. EEG provides millisecond-level resolution of early sensory processes, which is not feasible with fMRI studies. This prior study focused on the visual evoked potential (VEP) component called N2pc, which emerges under a visual search task at posterior electrode sites contralateral to an attended location at a latency of approximately 200–300 ms^10,11^. Trait-anxious participants exhibited enhanced N2pc responses when a visual distractor appeared simultaneously and in close spatial proximity to a visual target within a visual hemifield^9^. Although this finding appears to be consistent with the proposition that filtering out or inhibiting the task-irrelevant stimulus is impaired through early sensory processing in anxiety-prone individuals, it was difficult to disentangle neural activity from the distractor and the target in the previous N2pc study, as discussed previously^11^. Therefore, whether anxiety-related impairment of inhibition occurs during early sensory processing remains unclear. Considering that the inhibition function is expected to be pervasive across sensory modalities, one way to selectively scrutinize inhibition and its degradedness is to adopt a stimulation method whereby task-relevant and irrelevant stimuli are delivered in different sensory modalities. Neural activity relevant to early sensory processing of task-irrelevant stimuli and its modulation would then be discriminatingly evaluated by measuring early neural activity originating from the sensory modality of the task-irrelevant stimuli.

An EEG study using an auditory oddball paradigm revealed that the auditory evoked potential (AEP) in response to an abruptly presented infrequent stimulus (deviant) embedded in a train of frequent stimuli (standard) was negatively enhanced compared to that of frequent stimuli^12,13^, as both the deviant and the standard were task-irrelevant. This negative shift in the AEP, known as mismatch negativity (MMN), is quantified by subtracting the AEP of the standard from that of the deviant. The MMN appeared at a latency of approximately 100–200 ms, and its activity source was reported to lie around the auditory cortex^14–16^, which has been interpreted as reflecting pre-attentive detection of auditory changes based on sensory memory, in which a preceding sequential regularity of auditory events is formed^13,17^. In terms of sensory inhibition/restriction of task-irrelevant stimuli, previous studies evaluated MMNs^18–21^, but there was inconsistency in findings across these studies. With an increase in attentional demand, MMNs were reported to diminish^18,19^, and this finding was interpreted to reflect weakened detection of changes in task-irrelevant stimuli, originating from the attenuation of sensory processing of these stimuli. In other studies^20,21^, on the contrary, an increase in attentional demand was accompanied by enlargement in MMNs. The enlargement of MMNs was suggested to stem from the buildup in the detection of task-irrelevant stimuli due to an impairment of sensory inhibition for these stimuli; this impairment was supposed to originate from an overload in executive cognitive control. Meanwhile, MMNs were also shown to be insusceptible to top-down-mediated modulatory processes, such as those related to attentional load^22–26^.

Given these prior studies reporting various effects of attentional load on MMNs, it is possible that the inconsistency in findings may be attributed to the lack of consideration of individual traits, namely trait anxiety. If MMNs capture the anxiety-related impairment of inhibition, the reduction in MMNs due to increased attentional demand would be less pronounced, or the enlargement of MMNs could occur as an overall elevation of trait anxiety across participants. Such dispersing effects of trait anxiety on MMNs may have contributed to the inconsistent findings in the previous MMN literature. To elucidate whether anxiety-related impairment of inhibitory function impinges on early sensory processing, the present study presented task-irrelevant auditory stimuli under the oddball paradigm while participants performed a visual task. We hypothesized that enhancement of MMN with increasing attentional demands of the visual task, reflecting impaired inhibition of task-irrelevant stimuli, would be associated with trait anxiety.

In addition to the MMN, auditory deviants in the oddball paradigm are known to invoke a neural oscillation termed mismatch oscillatory response (MOR), typically in the theta–alpha band (4–12 Hz). The MOR is characterized by increased EEG power and/or greater phase alignment elicited by the deviant over the standard at an early latency range, including the MMN latency range^27,28^. In another research field, enhancement of neural oscillation in the alpha band, a part of the MOR frequency range, has been suggested as a neural index representing the sensory suppression of task-irrelevant sensory information^29,30^. As conjectured previously^31^, alpha oscillations induced by the deviant are expected to be modulated by the demands of behavioral tasks. Because the deviant in the oddball paradigm is task-irrelevant, inhibitory processing toward the deviant is expected to occur to some extent, and the increase in this inhibition—relative to that for the standard—would be expressed as an enhancement of MOR at the alpha band (hereafter, MOR‑α). From this perspective, we hypothesized that anxiety-related impairments in inhibitory processing would be reflected as a reduction in MOR-α power. To test this, the present study further examined whether decrements in MOR-α power would be associated with trait anxiety when the task became attentionally demanding.

## Methods

### Participants

Twenty healthy volunteers (16 males and 4 females; age 21–25 years, mean ± SD, 22.3 ± 1.16 years) participated in this study. All participants were right-handed and had normal visual acuity. They were all undergraduate or graduate students recruited at the Tokyo University of Science. Prior to their participation, all were provided with abstract information on the purpose of the present study such that this study was about attentional mechanisms. Informed consent was obtained from all participants. This study was conducted in accordance with the Declaration of Helsinki and was approved by the Ethics Committee of the Tokyo University of Science.

The data obtained from three participants were excluded from all analyses. The exclusion criterion was that the number of artifact-free EEG trials was below 50 trials for at least one condition (combination of stimulus categories (deviant/standard), the tone-frequencies, and the task difficulties).

### Stimulus and behavioral tasks

**Fig. 1:**
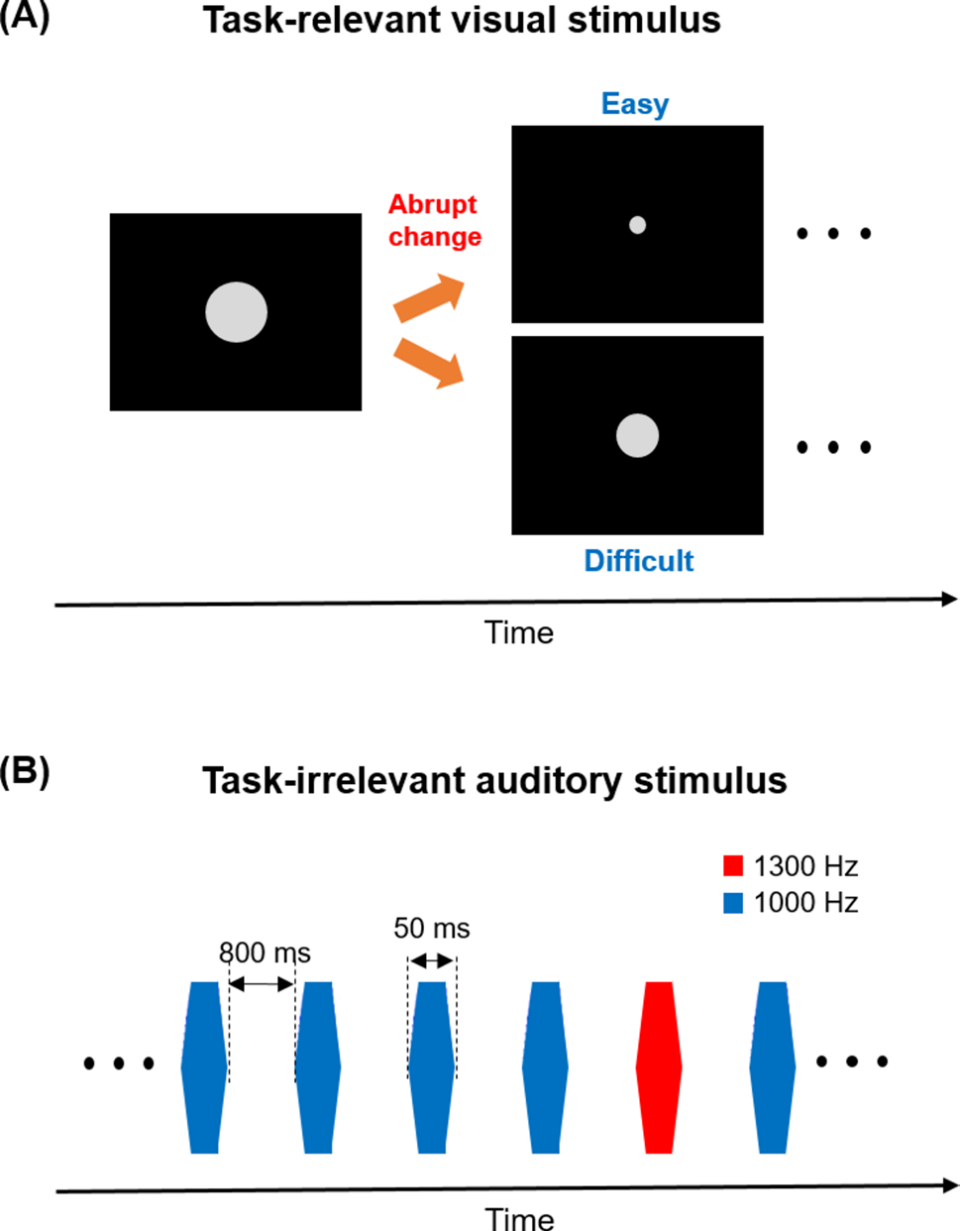
Time course of the stimulus presentation. (**A**) Task-Related Visual Stimuli. During the experiment, a white disc appeared at the center of the display and abruptly changed in size. Participants were asked to respond to the change as quickly and accurately as possible by pressing a keyboard. The magnitude of change in disc size was large for the easy condition and small for the difficult condition. Participants were expected to detect the change easily in the easy condition, whereas detection of the size change in the disc was expected to be more difficult in the difficult condition. Stimulations for easy and difficult conditions were implemented separately in different sessions. From the beginning of each task difficulty session, the disc size first decreased and then increased and decreased. At the beginning of every session, the words “Difficult task” and “Easy task” were presented for the difficult and easy conditions, respectively. (**B**) Task-Irrelevant Auditory Stimuli. The 1300 Hz and 1000 Hz tones were presented in an oddball paradigm, and the deviant and standard were presented at a probability of 20% and 80%, respectively. To obtain the MMN for every tone frequency, the mapping of the tone frequency to the deviant or standard was reversed in half of all sessions for each participant.

The stimulation paradigm is illustrated in Fig. 1A for visual stimulus and in Fig. 1B for auditory stimuli, respectively. The stimuli used in this study were created using MATLAB Psychophysics Toolbox (Brainard, 1997; Pelli, 1997; Kleiner et al., 2007). For visual stimulation, images were presented on a liquid crystal display (BENQ XL2540) at a refresh rate of 240 Hz. This study had two task-difficulty conditions—easy and difficult—each of which had four sessions. The words “Easy task” were centrally presented on the display at the beginning of each session of the easy condition, and the words “Difficult task” were presented in the same manner as in the difficult condition. In this manner, participants were informed of the condition of task difficulty. The presentation of the words lasted until the participants pressed the down arrow key on the keyboard in front of them. Following the keypress, a white disc with a diameter of 1.32° immediately and continuously appeared on the black background at the center of the display in both task-difficulty conditions. The luminance of the white disc was 5.86 cd/m^2^, and that of the background was 0.04 cd/m^2^. As previously suggested in ACT literature^2^, it is important to employ conceptually simple tasks to avoid intricate interpretations of the underlying mechanism. The disc abruptly changed in diameter to 1.25° for the difficult condition or to 0.81° for the easy condition, and the participants were required to respond to the change in disc size as quickly as possible. The magnitude of the change in disc size was smaller in the difficult condition than in the easy condition; detection of the size change in the disc was expected to be easier in the easy condition and more difficult in the difficult condition. There were 45 occurrences of disc size change in each session for each task-difficulty condition. The mean time interval between the changes in disc size was 5.69 ± 3.35 s (SD) across sessions, task-difficulty conditions, and participants. Each task-difficulty condition (easy or difficult) consisted of four sessions, with eight sessions for each participant. Eight sessions were randomized in sequence, regardless of the task difficulty, and participants were allowed to rest between sessions if needed. The completion of one session took approximately 4 to 5 min, excluding the rest period.

For both task-difficulty conditions, task-irrelevant auditory stimuli were presented to each participant. An oddball paradigm was employed for auditory stimulation, and two pure tones were delivered binaurally through headphones (Beyerdynamic DT1770 Pro). One of the two tones was 1300 Hz and the other was 1000 Hz. The duration of each presented tone was 50 ms, including linear rise and fall times of 10 ms each, and the inter-stimulus interval was set at 800 ms. The 1000 Hz tone was presented at 69.7 dB, and the peak amplitude of the 1300 Hz tone was the same as that of the 1000 Hz tone. In half of the sessions for each task difficulty (i.e., two sessions), the 1300 Hz tone and 1000 Hz tone were presented pseudo-randomly at probabilities of 20% and 80%, respectively; the former infrequently presented tone was the deviant (the 1300 Hz deviant), and the latter was the standard (the 1000 Hz standard) under the oddball paradigm. In the other half of the sessions, the mapping of tone frequency to the deviant or standard was reversed, such that the 1000 Hz tone was the deviant (the 1000 Hz deviant) and the 1300 Hz tone was the standard (the 1300 Hz standard). There were 300 tone presentations in each session, with 60 deviants and 240 standards per session. There were three constraints on the presentation of the tones. First, the standard tone was presented six times at the beginning of each session for each task-difficulty condition. Second, the deviant tone was not presented at the onset of the change in the size of the white disc. Third, at least three standards were inserted between the two closest deviants. Before the experiments, participants were informed that all the tones they would hear during the experiments were fully task-irrelevant and were further asked to concentrate on their behavioral task (detection of the change in disc size). The presentation of the auditory stimuli and that of the task-relevant visual stimuli were independently controlled. In the MMN literature, continuous visual tasks similar to the one employed in the present study have been widely used within the oddball paradigm.

### Measurements of trait anxiety

In this study, trait anxiety, a predisposition to anxiety, was assessed in each participant using the Japanese version of the State-Trait Anxiety Inventory (STAI) [Ref.^32^; for the original version^33^]. The Japanese version of the STAI has been standardized and widely employed. For the trait part of the STAI (ensuing STAI-T score, an index of predisposition to anxiety), Cronbach’s *α* was 0.92 in the present data.

### Analysis of behavioral data

The proportion of trials in which participants responded to the changes in disc size was calculated for each tone frequency pair (1300 Hz deviant/1000 Hz standard or 1000 Hz deviant/1300 Hz standard) for every task difficulty condition. In the calculation of the proportion, the number of trials in which participants responded to the change was divided by the total number of trials in each tone frequency pair for every task difficulty condition. Additionally, we calculated the mean reaction time (RT) to the onset of size changes in the disc size for each frequency pair for each task difficulty condition. The proportion and mean RT were assessed using a repeated-measures two-way analysis of variance (ANOVA) with tone frequency and task difficulty.

In addition, we evaluated whether behavioral performance would be affected by trait anxiety when the behavioral task became attentionally demanding. The differential proportion was calculated for each participant by subtracting the proportion in the easy condition from that in the difficult condition. Similarly, the differential RT (mean RT for the difficult condition—mean RT for the easy condition) was obtained. Then, the Pearson correlation coefficient between each of these two indices and the STAI-T scores across participants was evaluated. Correlation analysis of the differential proportion or differential RT was performed for each tone frequency pair. If there was any significant correlation, the significance level in the correlation analyses was controlled by the false discovery rate (FDR: *q* = 0.05) based on a prior study^34^. Hereafter, the *p*-value—corrected using the FDR—is expressed as *adj_p*.

### EEG recording

Neural activity was recorded using an electroencephalography (EEG) processor with 57 electrodes placed on the scalp (EEG-1200; Nihon Kohden, Tokyo, Japan; EasyCap GmbH, Herrsching, Germany). The impedance at each electrode was maintained below 10 kΩ. EEG signals were digitized at 1 kHz and recorded with a 0.5–300 Hz band-pass filter. EEG signals were referenced to the right earlobe during data acquisition.

### AEP analysis

The EEG signals were offline low-pass filtered at 30 Hz. EEG epochs were extracted from 100 ms before to 500 ms after the onset of the deviant or standard. EEG epochs containing a deflection greater than ± 100 μV in at least one electrode were excluded. To obtain AEPs, the remaining EEG epochs were averaged for each task-difficulty condition and tone frequency. The mean number of epochs averaged 99.67 ± 16.87 (SD) for the deviant and 316.03 ± 55.24 (SD) for the standard across the tone frequencies. The mean amplitude for a period of -100 to 0 ms relative to stimulus onset was used as the baseline. The AEPs obtained were referenced to the mean AEPs of the left and right mastoids. For each tone frequency, MMN was calculated by subtracting the AEP of the standard from that of the deviant. The AEP and MMN, which were recorded at Fz, were assessed in the analysis of evoked potentials. The MMN typically emerges over frontocentral electrode sites^35^, and it was reported to become particularly prominent at Fz when participants perform a visual task^24^. Peak latency and amplitude of the first prominent MMN were evaluated for each participant within a latency range of 100 to 250 ms. Data on peak latency and amplitude were submitted to a repeated-measures two-way ANOVA with tone frequency and task difficulty as factors. As in the behavioral analysis, we further evaluated whether the effects of an increase in task difficulty on MMN would depend on trait anxiety. For each participant, the differential peak latency or amplitude of the MMN was calculated for each tone frequency by subtracting the MMN peak latency or amplitude under the easy condition from that under the difficult condition. Pearson correlation coefficients between the differential latency and amplitude of the MMN (difficult-to-easy) and STAI-T scores were obtained across participants. In the correlation analysis for MMN regarding differential latency or amplitude, the significance level was controlled by the FDR (*q* = 0.05).

### Analysis of the time–frequency response

The EEG signals were re-referenced to the average of the signals recorded at left and right mastoids. A complex Morlet wavelet convolution was then performed on the raw EEG data to obtain the time–frequency response (TFR). The frequencies extracted in the TFR analyses ranged from 1 to 50 Hz with a 0.5 Hz linear step, and the length of the wavelet was linearly changed from two cycles at the lowest frequency to seven cycles at the highest frequency. Following this implementation of convolution, TFRs with trials of artifact-free epochs from  −400 ms before to 700 ms after the onset of the deviant or standard were collected for further analyses; the criterion to identify artifact-free data was the same as that utilized in the AEP analysis. Using the collected TFRs, the power values for the deviant and standard conditions were calculated and averaged across trials for each tone-frequency pair in each task difficulty condition. Baseline normalization was performed by decibel (dB) conversion with a baseline period of -400 to 0 ms prior to the onset of the deviant or standard. Furthermore, an analysis of inter-trial phase clustering (ITPC) was conducted (e.g., Cohen, 2014) to measure the extent to which the phase angles at each time–frequency point across trials are non-uniformly distributed.

Prior studies on MORs reported that EEG power and/or the degree of phase alignment across trials significantly increased in the theta band or theta-alpha band following the onset of the deviant over that of the standard at fronto-central electrode sites^27,28^. Based on these findings, we initially evaluated whether the deviant would enhance power and ITPC over the standard in the theta or theta-alpha band under the current experimental paradigm, as previously reported. After selecting the frontal 24 electrode sites (Fp1, Fpz, Fp2, AF7, AF3, AFz, AF4, AF8, F7, F5, F3, F1, Fz, F2, F4, F6, F8, FC5, FC3, FC1, FCz, FC2, FC4, and FC6), the mean power and ITPC across the electrodes for the deviant or standard in each tone frequency for each task difficulty were calculated. Subsequently, the mean power and ITPC were averaged across frequencies of 4–12 Hz (theta-alpha band) and latencies of 100–300 ms; the determination of electrodes to be selected and that of the time–frequency window were based on prior studies^27,28^. In the statistical analysis, the averaged power and ITPC values within the time–frequency window were then subjected to a repeated-measures three-way ANOVA with the factors of stimulus difference (deviant or standard), tone frequency, and task difficulty.

### Cluster-based permutation analysis for MOR

Similar to the MMN analysis, this study also assessed whether differential MOR (difficult-to-easy) correlated with trait anxiety scores across participants. In this analysis, focus was placed on a more spatially localized peak of the MOR on the scalp than in the MOR analysis described above. At the beginning of this analysis, two conditions of task difficulty (easy or difficult) were collapsed, and an electrode of interest indicating the most prominent MOR (power and ITPC) for each tone frequency was defined as follows: (1) Power and ITPC for the deviant were subtracted from those for the standard at each electrode throughout the whole time–frequency window (frequency: 1 to 50 Hz, latency:  −400 to 700 ms), yielding the power/ITPC of the collapsed task difficulty condition; (2) The power/ITPC of the MOR was averaged across all electrodes and participants, giving rise to the grand-global MOR power/ITPC. This grand-global MOR power/ITPC was obtained to initially evaluate the frequency band and latency of the MOR power/ITPC, which prominently appeared across all electrodes and participants in the current experimental paradigm; (3) The most prominent first local peak of the grand-global MOR power/ITPC was identified within the entire time–frequency window, and the frequency band at which the prominent peak emerged was determined by the full width at half maximum. For the 1300 Hz MOR, the identified frequency band of the prominent peak was 5 to 12 Hz (theta-alpha band) for power and 4.5 to 17 Hz (theta-low beta band) for ITPC. As for the 1000 Hz MOR, the identified frequency band of the prominent peak was 5 to 10.5 Hz for power and 4.5 to 11.5 Hz for ITPC; both frequency bands were within the theta-alpha band; and (4) At the peak latency for the mean of the grand-global MOR power/ITPC across the frequencies determined at the preceding step, the electrode of interest that indicated the most prominent power/ITPC was selected; all peak latencies of the prominent peaks for power/ITPC were within a range of about 190 to 300 ms across tone frequencies. The electrode of interest determined following the procedures above was expected to contribute the most to the emergence of the power/ITPC of the MOR across participants. For the 1300 Hz MOR, the electrodes of interest for power and ITPC were FC6 and F8, respectively. Regarding the 1000 Hz MOR, the electrode of interest for power was FCz, and that for ITPC was F6. All electrodes selected as the electrodes of interest were part of the frontal 24 electrodes that were employed in the first MOR analysis (see above). The current locations of the electrodes of interest on the scalp were consistent with prior MOR studies in that the electrode with the most prominent power or ITPC of the MOR was located around the central midline or the right side of the frontal electrode region^27,28^.

After selecting the electrode of interest, a correlation analysis was conducted to determine whether differential power/ITPC (difficult-to-easy) would correlate with STAI-T scores across participants using a non-parametric cluster-based permutation test^36,37^. This statistical test was performed for each 1300 Hz MOR and 1000 Hz MOR. The Pearson correlation coefficient between power or ITPC and STAI-T scores was calculated for each time–frequency point across the entire time–frequency window. Contiguous time–frequency points with statistical significance for the correlation were then clustered (pre-cluster threshold α = 0.05), and the sum of absolute t-values in each cluster was calculated for further evaluation regarding the statistical significance of the cluster’s emergence, in which a t-value was calculated in each correlation coefficient computation at one time–frequency point. To determine a cluster with statistical significance, the mapping of the STAI-T score to power or ITPC was shuffled 1000 times. The largest summed t-value across clusters in each shuffle was stored, and the null distribution of the largest summed t-values was obtained. Among all the original clusters extracted prior to shuffling, any cluster with a summed t-value exceeding the 95th percentile of the null distribution was considered statistically significant. Data are reported as the mean ± SE throughout the manuscript, except when noted otherwise.

## Results

### Behavioral data

**Fig. 2:**
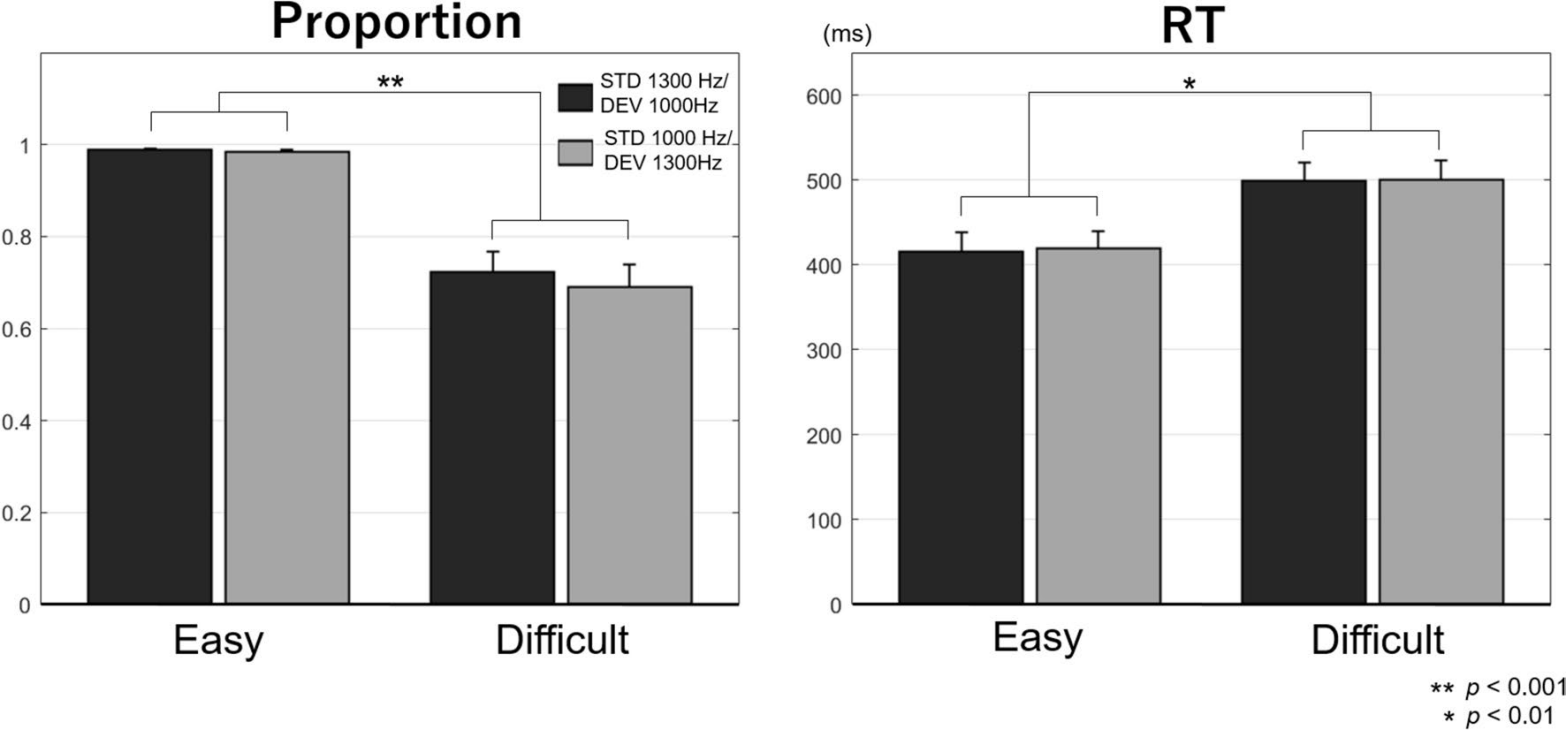
Mean behavioral data for every experimental condition. (**A**) Mean proportion of trials in which participants responded to abrupt changes in disc size. The proportion of participants in the easy condition was significantly higher than that in the difficult condition. (**B**) Mean RT for abrupt changes in disc size. The RT for the change in disc size in the difficult condition significantly increased compared with that in the easy condition.

Figure 2 shows the mean proportion of trials in which participants responded to an abrupt change in disc size and the mean RTs across participants. The mean proportion of responses to the change in disc size was significantly higher in the easy condition than in the difficult condition (main effect of task difficulty: *F* (1, 16) = 39.88, *p* < 0.001, ${\eta }_{p}^{2}$=0.71), indicating that it was more difficult for participants to detect the change in disc size in the difficult condition than in the easy condition in this study. There was no significant difference in the mean proportion between the tone frequency pairs (1300 Hz deviant/1000 Hz standard and 1000 Hz deviant/1300 Hz standard), and the interaction between task difficulty and tone frequency was not significant (main effect of the tone frequency pair: *F* (1, 16) = 2.81, *p* = 0.112, ${\eta }_{p}^{2}$=0.14; interaction between the two factors: *F* (1, 16) = 2.04, *p* = 0.172, ${\eta }_{p}^{2}$=0.11). Regarding the mean RT to the change in disc size, there was a significant elongation for the difficult condition compared to the easy condition (main effect of task difficulty: *F* (1, 16) = 45.33, *p* < 0.001, ${\eta }_{p}^{2}$=0.73). Meanwhile, the mean RT was not significantly affected by tone frequency (*F* (1, 16) = 0.16, *p* = 0.686, ${\eta }_{p}^{2}$=0.01), and there was no significant interaction between task difficulty and tone frequency (*F* (1, 16) = 0.14, *p* = 0.707, ${\eta }_{p}^{2}$=0.009). In concert with the higher proportion in the easy condition over the difficult condition, this significant increase in the mean RT supports the validity of the current experiment in elevating task difficulty in the difficult condition compared to the easy condition.

**Fig. 3:**
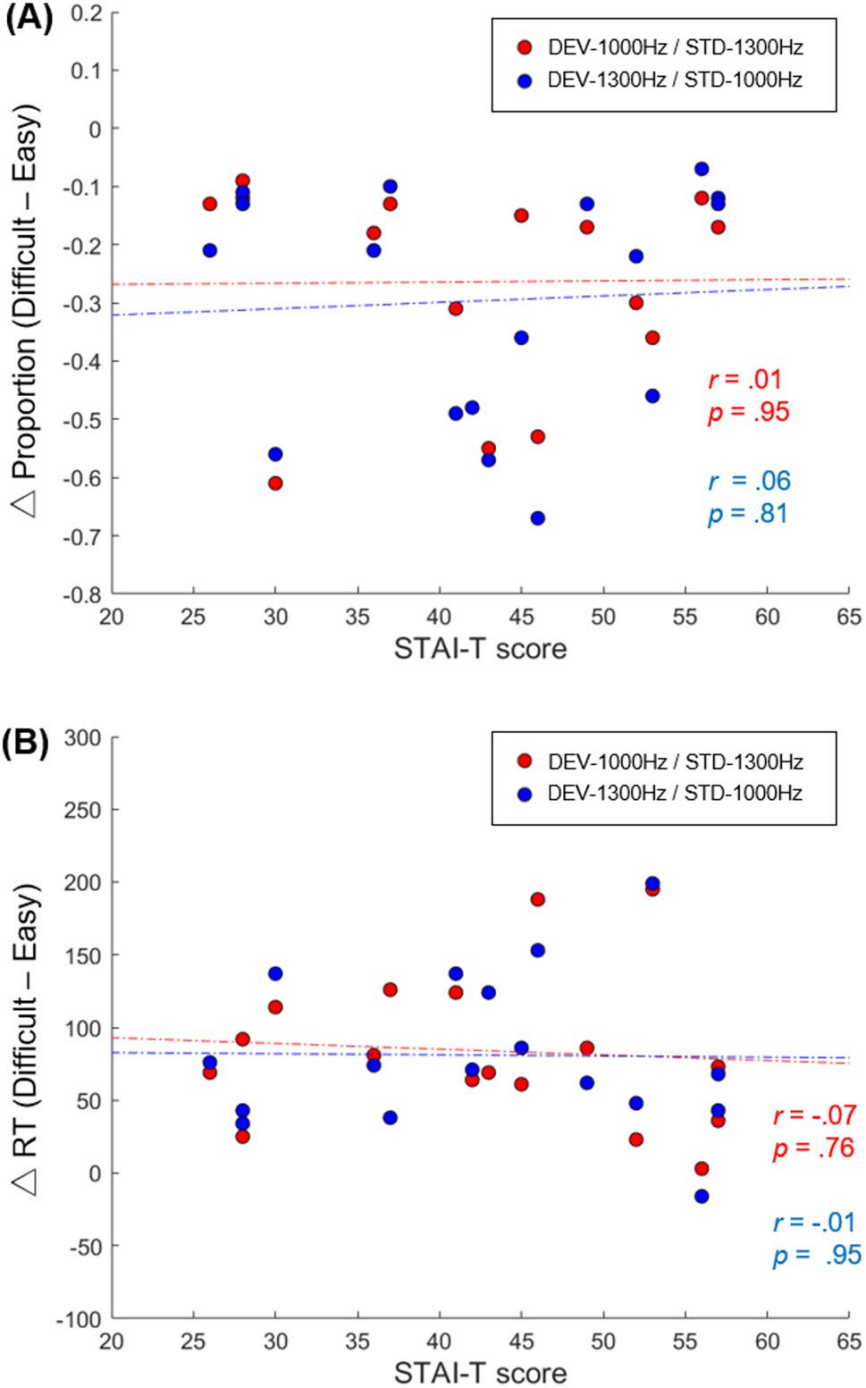
Relationship between trait anxiety and behavioral data. (**A**) Relationship between trait anxiety and differential proportions (difficult-to-easy) across participants. There was no significant correlation between the STAI-T score and the differential proportion for the DEV-1000 Hz/STD-1300 Hz pair (red circle) and the DEV-1300 Hz/STD-1000 Hz pair (blue circle). (**B**) Relationship between trait anxiety and differential RT (difficult-to-easy) across participants. The STAI-T score did not significantly correlate with differential RT across participants for either the DEV-1000 Hz/STD-1300 Hz pair (red circle) or the DEV-1300 Hz/STD-1000 Hz pair (blue circle).

In the present study, the STAI-T scores ranged from 26 to 57 across participants (mean ± SD, 42.7 ± 10.6). As a behavioral task becomes attentionally demanding, a higher level of anxiety is expected to impair behavioral performance to some extent^1,3^. As shown in Fig. 3A, there was no significant relationship between the STAI-T score and the differential proportion (difficult-to-easy) across participants for each tone frequency pair (for the 1000 Hz deviant/1300 Hz standard, *r* = 0.01, 95% CI [−0.47, 0.49], *p* = 0.958; for the 1300 Hz deviant/1000 Hz standard, *r* = 0.06, 95% CI [−0.43, 0.52], *p* = 0.818). Furthermore, the STAI-T score was not significantly correlated with the differential RT (difficult-to-easy) across participants for either tone frequency pair (for 1000 Hz deviant/1300 Hz standard, *r* = -0.07, 95% CI [−0.53, 0.41], *p* = 0.765; for 1300 Hz deviant/1000 Hz standard, *r* = -0.01, 95% CI [−0.49, 0.46], *p* = 0.951) (Fig. 3B). Collectively, effects of trait anxiety on behavioral performance were not emergent at the behavioral level under the current experimental paradigm.

### AEP and MMN data

**Fig. 4:**
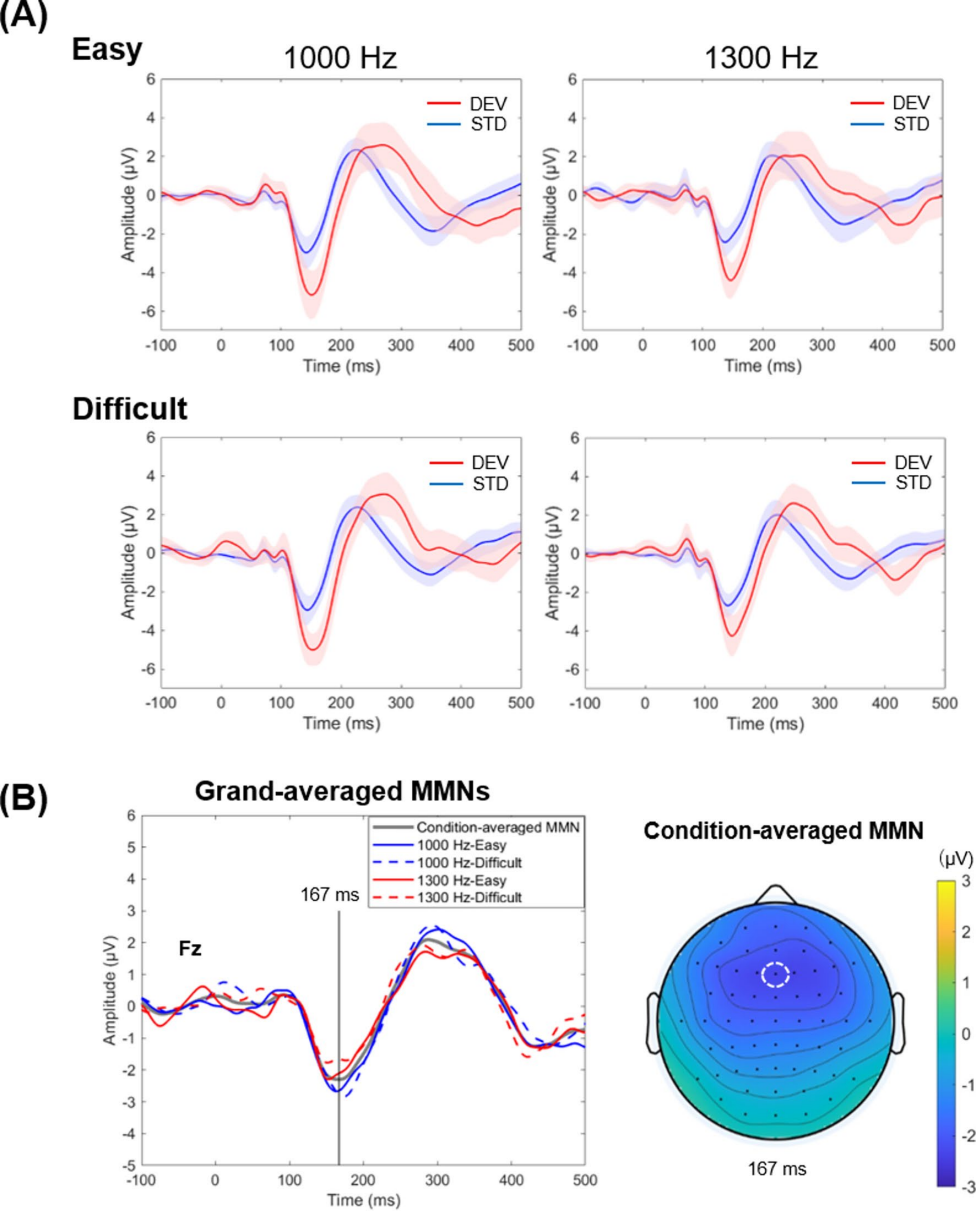
AEPs and MMNs. (**A**) Grand-averaged AEP in every task difficulty condition and for each tone frequency. The average AEP for the deviant is depicted in red and that for the standard is depicted in blue; the 95% confidence interval is also illustrated in the respective color. In every task difficulty condition for each tone frequency, a prominent N1 appeared at a latency of approximately 150 ms for both the deviant and standard, and N1 was more pronouncedly evoked for the deviant compared with the standard. (**B**) Grand-averaged MMN in every task difficulty condition as well as for each tone frequency (left panel), and isocontour map of the condition-averaged MMN. The MMN emerged at a latency of approximately 170 ms at Fz. The MMN prominently and symmetrically appeared at Fz across task difficulty conditions and tone frequencies.

Figure 4A shows the grand-averaged AEPs time-locked to the onset of the deviant or standard tone frequency for each task difficulty condition. For all tone frequencies and task difficulty conditions, the first prominent negative potential, N1, appeared at a latency of approximately 150 ms, and the AEP evoked by the deviant was markedly larger than that evoked by the standard, confirming the presence of a clear MMN across all experimental conditions. Figure 4B shows the grand-averaged MMNs at Fz for all tone frequencies and task difficulty conditions (left panel) as well as an isocontour map of the condition-averaged MMN (the mean of the grand-averaged MMNs across all experimental conditions) (right panel). MMN appeared at a latency of approximately 170 ms at Fz across participants under all experimental conditions (mean latency, 171.2 ± 24.5 (SD)). Statistical analyses revealed that neither the tone frequency nor the task difficulty condition significantly affected MMN peak latency (main effect of tone frequency: *F* (1, 16) = 0.002, *p* = 0.964, ${\eta }_{p}^{2}$=0.0001; main effect of task difficulty: *F* (1, 16) = 0.68, *p* = 0.419, ${\eta }_{p}^{2}$=0.04) or its peak amplitude (main effect of tone frequency: *F* (1, 16) = 1.96, *p* = 0.180, ${\eta }_{p}^{2}$=0.10; main effect of task difficulty: *F* (1, 16) = 0.04, *p* = 0.830, ${\eta }_{p}^{2}$=0.003). There was no significant interaction between tone frequency and task difficulty for MMN latency (*F* (1, 16) = 0.03, *p* = 0.849, ${\eta }_{p}^{2}$=0.002) or amplitude (*F* (1, 16) = 0.53, *p* = 0.473, ${\eta }_{p}^{2}$=0.03).

**Fig. 5:**
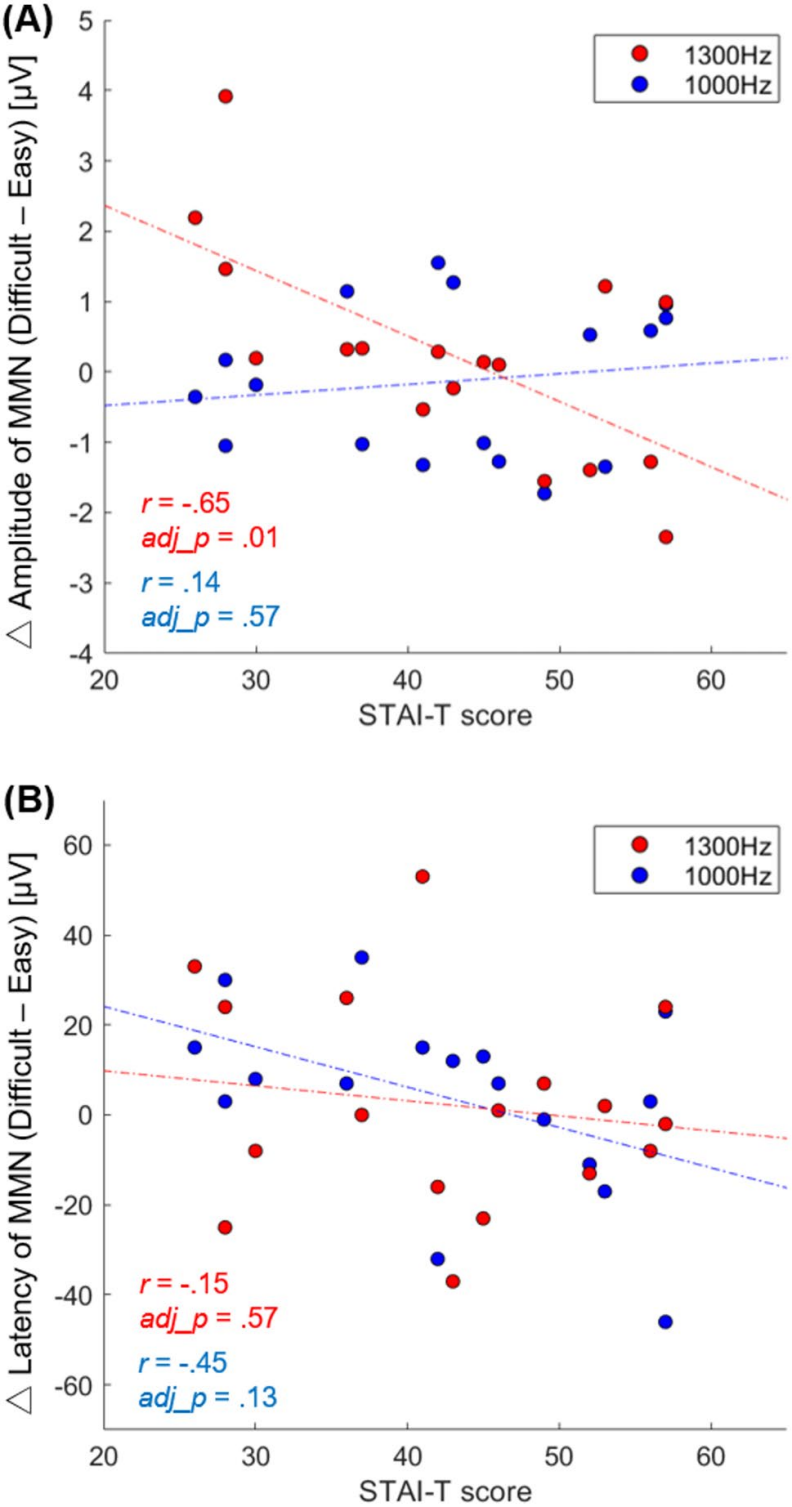
Relationship between trait anxiety and differential latency or amplitude of MMN (difficult-to-easy). (**A**) Relationship between trait anxiety and differential amplitude of MMN (difficult-to-easy) across participants. For the 1300 Hz tone, an increase in the STAI-T score was significantly correlated with enhancement of the differential amplitude of the MMN, indicating that the prediction error for the task-irrelevant tone was magnified as the STAI-T score increased when the behavioral task was taxed. For the 1000 Hz tone, there was no significant correlation between the STAI-T score and the differential amplitude of MMN. (**B**) Relationship between trait anxiety and differential latency of MMN (difficult-to-easy) across participants. The STAI-T score did not significantly correlate with the differential latency of the MMN for either the 1300 Hz or 1000 Hz tones.

Regarding inter-individual differences in predisposition to anxiety, we evaluated the relationship between the STAI-T score and the differential amplitude or latency of MMN (difficult-to-easy), as in the behavioral analysis. We found a significant correlation between the STAI-T score and the differential amplitude of the MMN across participants for a tone frequency of 1300 Hz (*r* = -−0.65, 95% CI [ −0.86,  −0.26], *adj_p* = 0.01), and the increase in the STAI-T score was significantly associated with a relative enhancement of the MMN amplitude for the difficult condition compared to the easy condition for the tone frequency of 1300 Hz (Fig. 5A). However, there was no significant relationship between these two indices for a tone frequency of 1000 Hz (*r* = 0.14, 95% CI [ −0.35, 0.58], *adj_p* = 0.57). Regarding the differential latency of the MMN, the STAI-T score was not significantly correlated with the differential latency across participants for either tone frequency (1300 Hz: *r* =  −0.15, 95% CI [ −0.58, 0.35], *adj_p* = 0.57; 1000 Hz: *r* =−  0.45, 95% CI [− 0.76, 0.03], *adj_p* = 0.13) (Fig. 5B). The findings for the 1300‑Hz tone support our hypothesis that anxiety‑related impairment of the inhibition function becomes prominent when the predisposition to anxiety is high. We further examined whether this anxiety‑related impairment for the 1300‑Hz tone would be sufficiently pronounced to be reflected in the MMN peak amplitude itself—particularly in the difficult condition—rather than only in the differential index between task‑difficulty conditions. However, statistical analyses revealed that this impairment was not significantly reflected in the MMN peak amplitude in either the difficult or the easy condition (see Supplementary Fig. S1 for detailed information, including related results).

### MOR data

**Fig. 6:**
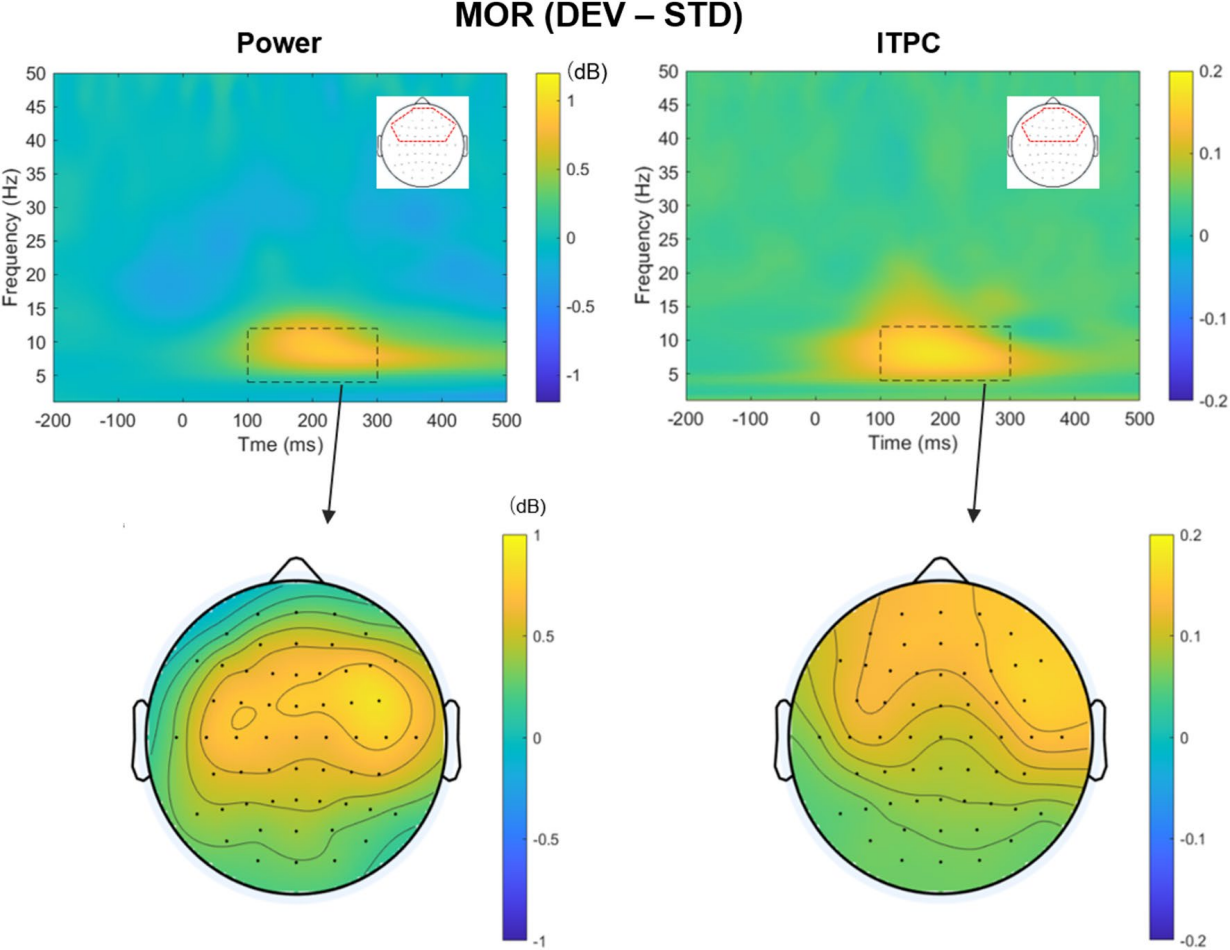
Mismatch oscillatory responses (MORs). The grand-averaged power (left panel) and ITPC (right panel) of the MOR at the fronto-central electrode area are shown, and the fronto-central electrode area defined in the current study is shown in the upper-right part of the time–frequency representation. The MOR power and ITPC clearly peaked within the time–frequency window of 100–300 ms and the theta-alpha band (4–12 Hz). Isocontour maps of the mean power and ITPC across the time–frequency window showed that the most prominent local peak emerged at the fronto-central electrode area.

This study evaluated whether EEG power and ITPC in the theta or alpha bands would increase following the onset of the deviant compared to the standard around the frontocentral electrode sites, as reported previously^27,28^. Figure 6 illustrates the grand-averaged power/ITPC of the MOR in the frontocentral electrode area. Statistical analysis revealed that the EEG power induced by the deviant was significantly larger than that induced by the standard within a time–frequency window of 100–300 ms and 4–12 Hz (mean power of the deviant: 0.302 ± 0.178 dB; mean power of the standard:  −0.275 ± 0.151 dB; main effect of stimulus difference: *F* (1, 16) = 36.042, *p* < 0.001, ${\eta }_{p}^{2}$=0.69), indicating a significant emergence of MOR power at the theta-alpha band under the current experiments (upper left panel of Fig. 6). The spatial peak of MOR power appeared to be lateralized to the right side of the frontal electrodes (lower left panel of Fig. 6), consistent with a prior study^27^. The enhancement of EEG power for the deviant over the standard (i.e., the emergence of MOR power) was not significantly affected by any other factor (stimulus difference x task difficulty: *F* (1, 16) = 0.176, *p* = 0.680, ${\eta }_{p}^{2}$=0.01; stimulus difference x tone frequency: *F* (1, 16) = 0.467, *p* = 0.503, ${\eta }_{p}^{2}$=0.02; stimulus difference x task difficulty x tone frequency: *F* (1, 16) = 0.282, *p* = 0.602, ${\eta }_{p}^{2}$=0.01). Additionally, EEG power was not significantly affected by task difficulty (mean power of the difficult condition: -0.052 ± 0.149 dB; mean power of the easy condition: 0.078 ± 0.192 dB; main effect of task difficulty: *F* (1, 16) = 1.311, *p* = 0.268, ${\eta }_{p}^{2}$=0.07), and the power induced by the 1300 Hz tone was significantly lower than that induced by the 1000 Hz tone (mean power of 1300 Hz tone:  −0.110 ± 0.181 dB; mean power of 1000 Hz tone: 0.136 ± 0.160 dB; main effect of tone frequency: *F* (1, 16) = 8.452, *p* = 0.010, ${\eta }_{p}^{2}$=0.34). There was no significant interaction between these two factors (task difficulty x tone frequency: *F* (1, 16) = 1.013, *p* = 0.329, ${\eta }_{p}^{2}$=0.05).

Concerning ITPC, it significantly increased for the deviant compared to the standard within a time–frequency window of 100–300 ms and 4–12 Hz (mean ITPC of the deviant: 0.368 ± 0.017; mean ITPC of the standard: 0.234 ± 0.013; main effect of stimulus difference: *F* (1, 16) = 105.954, *p* < 0.001, ${\eta }_{p}^{2}$=0.86), indicating a significant emergence of MOR phase alignment at the theta-alpha band in this study (upper right panel of Fig. 6). The ITPC enhancement for the deviant over the standard appeared in both tone frequency conditions, and the enhancement was significantly larger for the 1000 Hz tone than for the 1300 Hz tone (1000 Hz tone: mean ITPC of the deviant, 0.392 ± 0.017; standard, 0.243 ± 0.015; 1300 Hz tone: mean ITPC of the deviant, 0.343 ± 0.017; standard, 0.225 ± 0.012; stimulus difference x tone frequency: *F* (1, 16) = 5.911, *p* = 0.027, ${\eta }_{p}^{2}$=0.26; simple main effect of stimulus difference for 1000 Hz tone: *F* (1, 16) = 147.343, *p* < 0.001, ${\eta }_{p}^{2}$=0.90; simple main effect of stimulus difference for 1300 Hz tone: *F* (1, 16) = 51.652, *p* < 0.001, ${\eta }_{p}^{2}$=0.76). Further analyses revealed that the ITPC of the 1000 Hz tone significantly increased compared to that of the 1300 Hz tone for the deviant (simple main effect of tone frequency for the deviant: *F* (1, 16) = 17.034, *p* < 0.001, ${\eta }_{p}^{2}$=0.51). In contrast, there was no significant difference between the ITPC of the 1000 Hz tone and that of the 1300 Hz tone for the standard (simple main effect of tone frequency for the standard: *F* (1, 16) = 4.055, *p* = 0.061, ${\eta }_{p}^{2}$=0.20). Regardless of stimulus type, the ITPC of the 1300 Hz tone was significantly smaller than that of the 1000 Hz tone (mean ITPC of the 1300 Hz tone: 0.284 ± 0.018; mean ITPC of the 1000 Hz tone: 0.318 ± 0.020; main effect of tone frequency: *F* (1, 16) = 16.703, *p* < 0.001, ${\eta }_{p}^{2}$=0.51). Task difficulty did not significantly affect ITPC (*F* (1, 16) = 3.346, *p* = 0.086, ${\eta }_{p}^{2}$=0.17), and its interactions with other factors were not statistically significant (task difficulty x stimulus difference: *F* (1, 16) = 3.013, *p* = 0.101, ${\eta }_{p}^{2}$=0.15; task difficulty x tone frequency: *F* (1, 16) = 0.268, *p* = 0.611, ${\eta }_{p}^{2}$=0.01; stimulus difference x tone frequency x task difficulty: *F* (1, 16) = 0.337, *p* = 0.569, ${\eta }_{p}^{2}$=0.02).

**Fig. 7:**
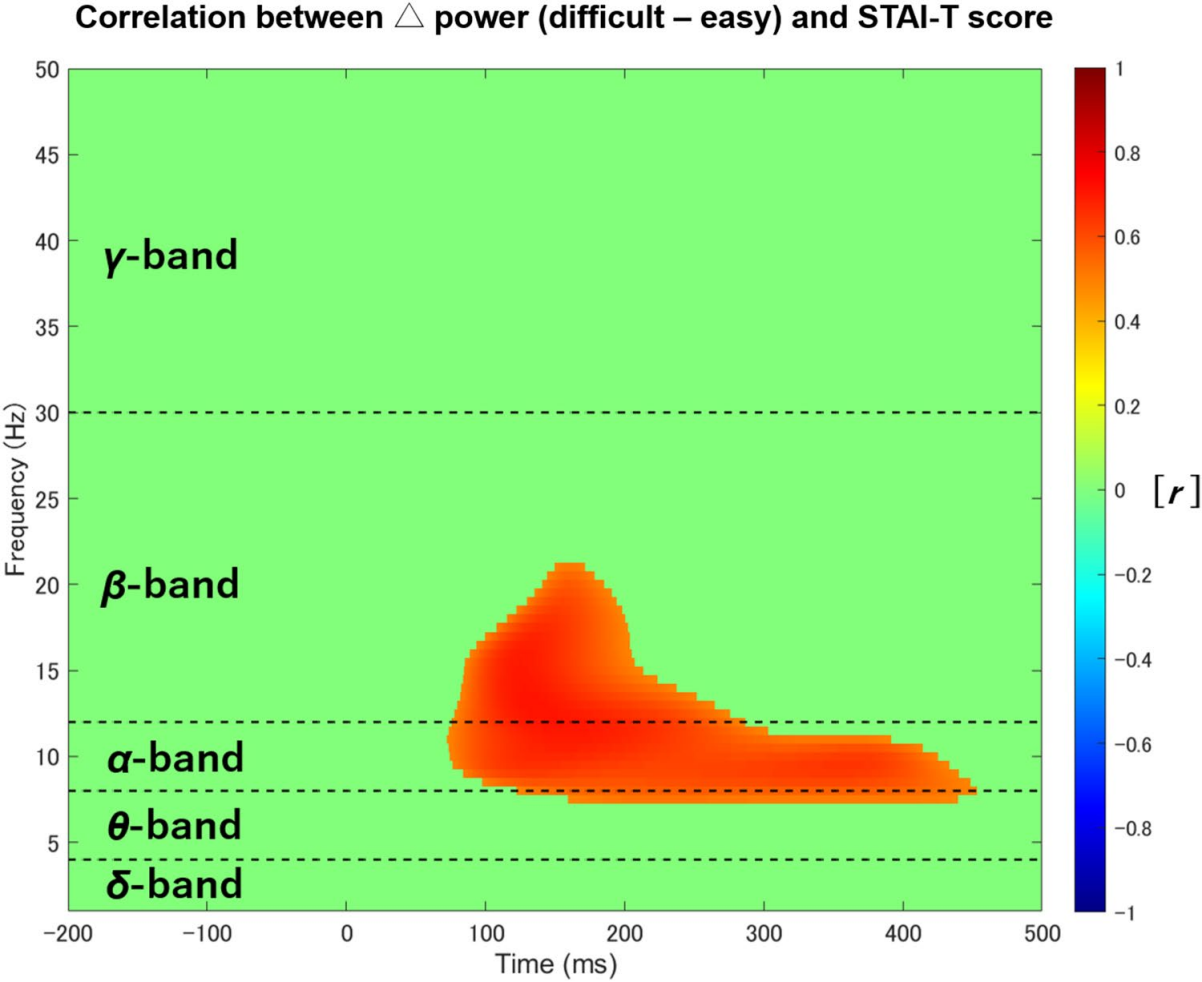
Correlation between trait anxiety and differential MOR power (Difficult-to-easy). Pearson’s correlation coefficient with statistical significance is illustrated on a time–frequency window, and the correlation between the STAI-T score and differential MOR power (difficult-to-easy) across participants was evaluated at each pixel of the time–frequency window using a nonparametric cluster-based permutation test (for details, see the Methods section). A significant emergence of clusters was observed, with a correlation between an increase in the STAI-T score and an enhancement of the MOR power. This significant cluster appeared over a range around the alpha band at latencies of approximately 100–450 ms and over the low beta band at latencies of approximately 100–200 ms.

As in the analysis of MMN, this study attempted to determine whether the increase in STAI-T scores would correlate with differences in MOR-α power (difficult-to-easy) across participants. A non-parametric cluster-based permutation test revealed one significant cluster in the entire time–frequency window for the 1300 Hz tone (Fig. 7); there was no significant cluster for the 1000 Hz tone. The significant cluster appeared at MOR-α’s latencies of approximately 90–450 ms, overlapping with the latencies of the MMNs. All pixels comprising the significant cluster indicated a positive correlation between the STAI-T score and the differential power of the MOR (difficult-to-easy). This finding contradicts this study’s hypothesis that susceptibility to task-irrelevant sensory change is associated with a reduction in MOR-α power when the task is attentionally demanding. Instead, this study’s findings indicate that susceptibility to task-irrelevant sensory change for anxious participants is reflected in the “augmentation” of MOR-α power when the task is attentionally demanding. As shown in Fig. 7, the significant cluster included neural oscillations at the low beta band as well as those at the alpha band. Overall, these findings regarding neural oscillation suggest that impaired top-down inhibition of task-irrelevant sensory change is reflected in the increased power of MOR-α and neural oscillation at the low beta band in anxious individuals. For ITPC of the MOR, no significant cluster was found in the whole time–frequency window set in this study.

## Discussion

Using the MMN, this study examined whether the anxiety-related impairment of inhibitory process toward task-irrelevant sensory changes is reflected in early sensory processing following the onset of a change. Supporting this study’s hypothesis, trait anxiety was significantly correlated with MMN enhancement under the difficult (attentionally demanding) condition compared to the easy (less demanding) condition. Additionally, by focusing on neural oscillation at an early latency range including MMN’s latency range, namely, the reduction of MOR-α power, which is presumed to reflect the degradation of top-down-mediated sensory suppression for sensory change, the hypothesis that trait anxiety is associated with the reduction of MOR-α power when the task is attentionally demanding was tested. A non-parametric cluster-based permutation test revealed the emergence of a cluster with a significant positive correlation between power enhancement and STAI-T score on TFR at the alpha band as well as at the low beta band when the task became attentionally demanding. In contrast to this study’s hypothesis, trait anxiety was associated with power enhancement in the alpha band. Power enhancements at the alpha/low beta band may capture the occurrence of additional sensory-suppression-related top-down processing toward task-irrelevant events other than the deterioration of sensory suppression.

Prior studies have evaluated whether task difficulty affects MMN^18–26^; however, the effects of task difficulty on MMN remain inconclusive. While MMN was reported to be insusceptible to top-down-mediated modulatory processes, such as processes related to attentional load^22–26^, attentional modulations of MMNs have been inconsistently reported; an increase in task difficulty diminished^18,19^ or enlarged MMN^20,21^. In contrast to the present study, prior studies did not consider trait anxiety. Interindividual differences in anxiety‑related impaired inhibition may therefore have obscured the effect of task difficulty on MMNs to some extent. Another factor contributing to this inconsistency may be differences in task paradigms across studies. The enlarged MMNs were reported using working memory tasks that imposed high cognitive control^20,21^. The use of tasks requiring high cognitive control may have increased the likelihood of overloading the inhibition function compared with simpler tasks. As a result, degradation of the inhibition function might have been pronounced under the tasks with high cognitive control, especially for anxiety-prone individuals, leading to overall enhancements of MMN across participants.

Event-related potentials (ERPs) other than MMN to a task-irrelevant stimulus have been well reported to diminish when ongoing task difficulty becomes high^38–40^; ERP components evoked by auditory stimuli, such as N1, P2, and P3, have been the focus. Such diminished ERPs in response to a task-irrelevant stimulus, which are assumed to mirror the amount of residual attentional resources while performing a main behavioral task, can be used as a neural index for evaluating mental workload. Individual traits were not considered when measuring mental workload using the aforementioned ERPs. Meanwhile, mental workload measurement using the MMN may be applicable for individuals with low trait anxiety but not for individuals with high trait anxiety (see Fig. 5A). With a focus on an individual trait, namely trait anxiety, this study’s findings raise the possibility that mental workload measurement can capture the effectiveness of attentional control, specifically inhibition function, rather than attentional resources, as long as MMN is used as an index.

The current findings on MMN and neural oscillations have been coherently interpreted using a predictive coding framework^41,42^. In this framework, a prediction error (PE) occurs because of the inconsistency between bottom-up sensory information and top-down predictions of cortical processing following the onset of sensory input. PE is supposed to occur at the pre-attentive level of neural processing and to ascend the cortical hierarchy, and it is minimized through an interaction between top-down and bottom-up processing throughout hierarchical cortical processing. By incorporating the predictive coding framework into the MMN literature, MMN has been emphasized to reflect the emergence of PE in relation to a preceding sequential rule of sensory events^41,43^, and its amplitude is expected to mirror the magnitude of PE, as previously assumed^44,45^. While a behavioral task is taxed under limited neural resources, the emergence of PE invoked by an abrupt sensory change should be appropriately mitigated through top-down-mediated inhibition processing to the extent that the sensory change is task-irrelevant; otherwise, failure of the appropriate mitigation of task-irrelevant PE would contribute to an adverse effect of the sensory change on the performance of an ongoing behavioral task. From this perspective, this study’s findings of a significant correlation between MMN amplitude and STAI-T score indicate a failure to mitigate task-irrelevant PE with the manifestation of individual traits of predisposition to anxiety. Conversely, predictive coding posits that neural oscillations at the alpha and beta bands reflect top-down processing related to prediction^42,46^ and are also related to the modulation of PE gain, dependent on task relevance^42,46,47^. The enhancement of neural oscillations in the alpha band may represent the sensory suppression of task-irrelevant sensory information^29,30^. Enhancement at the beta band was reported to occur in a participant’s anxious state during a reward-based learning task, suggesting that the enhanced oscillation at this frequency band would downweight the effects of PE on further neural processing^48^. Although the behavioral task and experimental paradigm employed in prior studies were different from those in this study, enhancements of MOR power at the alpha and beta bands with a preponderance of trait anxiety may reflect an emergence of top-down-mediated compensatory suppression toward task-irrelevant sensory events when task-irrelevant PE reflected in MMN failed to be attenuated, possibly because such compensatory suppression operates to mitigate the detrimental effects of task-irrelevant PE on the performance of a given behavioral task by abolishing or down-weighting the gain of the task-irrelevant PE. Intriguingly, this study’s findings appear to indicate that such neural processing related to compensatory suppression occurs pre-attentively, as early as 450 ms post-stimulus (see Fig. 7).

As shown in this study’s results, a significant association between trait anxiety and enhancement of MMN amplitude or MOR power at the alpha or low-beta band for the 1300 Hz tone, but not for the 1000 Hz tone, was found. When deviants were presented under the present scheme of stimulation, there was an increase in MMN to the 1300 Hz tone (a deviant tone at 1300 Hz occurred in a stream of intermittent standard tones at 1000 Hz) and a decrease in MMN to the 1000 Hz tone (a deviant tone at 1000 Hz occurred in a stream of intermittent standard tones at 1300 Hz). This difference in the direction of the tone’s frequency change may have led to incongruent findings between tone frequencies. A prior study demonstrated that MMN evoked by a higher tone frequency was more augmented than that evoked by a lower frequency^49^, suggesting an intrinsic susceptibility to higher-pitched tones. Although this study did not find a frequency-dependent MMN difference overall, it is possible that susceptibility to a higher-frequency tone could have been magnified for anxious individuals when the task became attentionally demanding.

## Supplementary Information

Supplementary Information.

## Data Availability

The datasets generated and analyzed in this study are available from the corresponding author upon reasonable request under a collaborative research agreement.
