# Supplementary material for: Anxiety-related impairment of inhibition during early sensory processing: an MMN/MOR study
Source: Sci Rep. 2026 Apr 29;16:19844. doi: 10.1038/s41598-026-49460-9 (PMC13316063; doi:10.1038/s41598-026-49460-9)
Supplement: Supplementary file 1 — Supplementary Information. [file 41598_2026_49460_MOESM1_ESM.pdf]

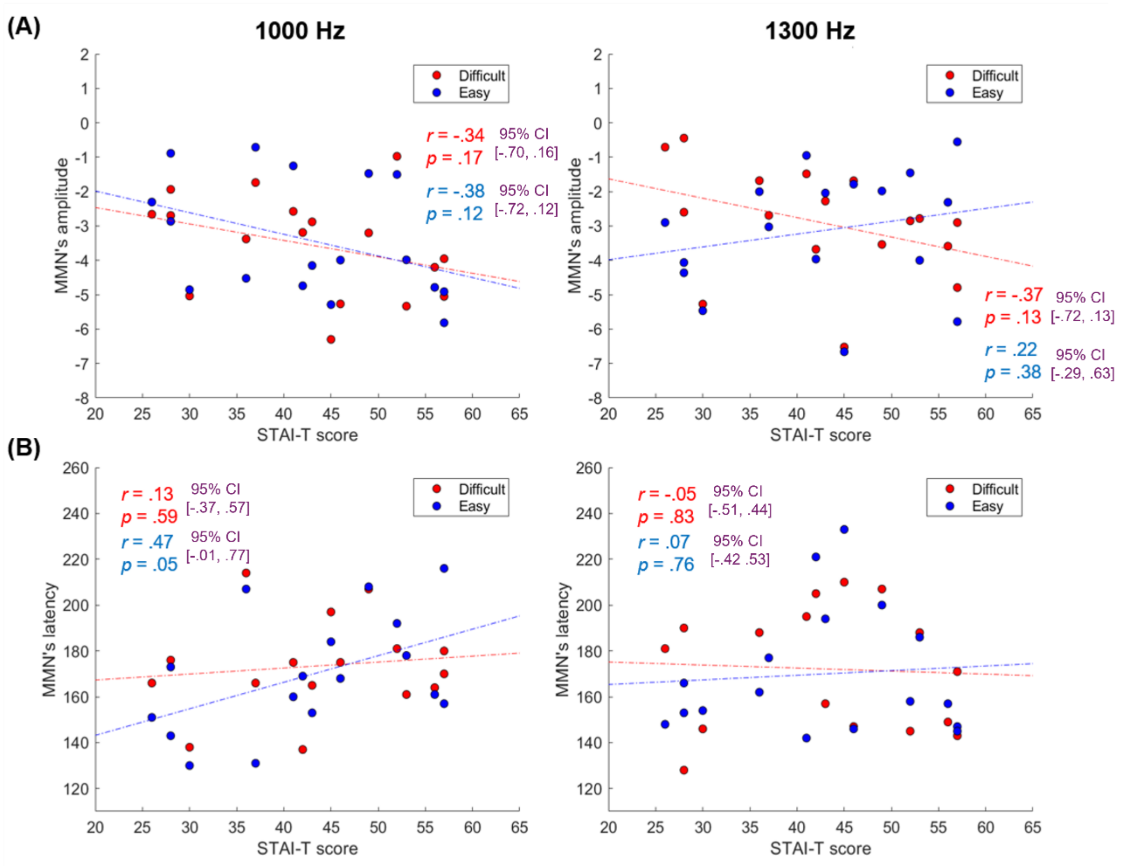

Fig. S1. Relationship between trait anxiety and MMN (non-differential MMN).

(A) Relationship between the STAI-T score and MMN amplitude across participants for the 1000 Hz (left panel) and 1300 Hz (right panel) tones. The STAI-T score was not significantly correlated with MMN peak amplitude for either tone frequency in both the difficult and easy conditions. These findings indicate that trait anxiety was not reflected in MMN amplitude.

(B) Relationship between the STAI-T and MMN latency across participants for the 1000 Hz (left panel) and 1300 Hz (right panel) tones. For both tone frequencies, there was no significant correlation between the STAI-T score and MMN peak latency in either task-difficulty condition. These results indicate that trait anxiety was not reflected in MMN peak latency.
